# Beta-Glucan Alters Gut Microbiota and Plasma Metabolites in Pre-Weaning Dairy Calves

**DOI:** 10.3390/metabo12080687

**Published:** 2022-07-26

**Authors:** Zhengzhong Luo, Li Ma, Tao Zhou, Yixin Huang, Liben Zhang, Zhenlong Du, Kang Yong, Xueping Yao, Liuhong Shen, Shumin Yu, Xiaodong Shi, Suizhong Cao

**Affiliations:** 1 Key Laboratory of Coarse Cereal Processing, Ministry of Agriculture and Rural Affairs, Chengdu University, Chengdu 610106, China; zhengzhongluo@163.com; 2 Department of Clinical Veterinary Medicine, College of Veterinary Medicine, Sichuan Agricultural University, Chengdu 611130, China; lima3697@163.com (L.M.); zhoutao0428@163.com (T.Z.); zhliben@163.com (L.Z.); dzl960730@163.com (Z.D.); yaoxueping74@126.com (X.Y.); shenlh@sicau.edu.cn (L.S.); yayushumin@sicau.edu.cn (S.Y.); 3 Institute of Biodiversity, Animal Health & Comparative Medicine, College of Medical, Veterinary & Life Sciences, University of Glasgow, Glasgow G61 1QH, UK; hyxemma213@gmail.com; 4 Department of Animal Science and Technology, Chongqing Three Gorges Vocational College, Chongqing 404100, China; yongkangkang@126.com

**Keywords:** beta-glucan, dairy calves, pre-weaning period, gut microbiota, plasma metabolites

## Abstract

The present study aims to evaluate the alterations in gut microbiome and plasma metabolites of dairy calves with β-glucan (BG) supplementation. Fourteen healthy newborn dairy calves with similar body weight were randomly divided into control (*n* = 7) and BG (*n* = 7) groups. All the calves were fed on the basal diet, while calves in the BG group were supplemented with oat BG on d 8 for 14 days. Serum markers, fecal microbiome, and plasma metabolites at d 21 were analyzed. The calves were weaned on d 60 and weighed. The mean weaning weight of the BG group was 4.29 kg heavier than that of the control group. Compared with the control group, the levels of serum globulin, albumin, and superoxide dismutase were increased in the BG group. Oat BG intake increased the gut microbiota richness and decreased the Firmicutes-to-Bacteroidetes ratio. Changes in serum markers were found to be correlated with the plasma metabolites, including sphingosine, trehalose, and 3-methoxy-4-hydroxyphenylglycol sulfate, and gut microbiota such as *Ruminococcaceae_NK4A214*, *Alistipes*, and *Bacteroides*. Overall, these results suggest that the BG promotes growth and health of pre-weaning dairy calves by affecting the interaction between the host and gut microbiota.

## 1. Introduction

The milk-feeding period is a key stage closely related to the growth and development of dairy calves. High pre-weaning growth rates and weaning weights contribute to the increased profitability of dairy cows during the production phase [1]. However, there are factors that can significantly affect the growth of pre-weaning dairy calves, such as nutritional imbalances, diseases, and dysbiosis of the gut microbiota [2,3,4]. There is increasing evidence that animal health and growth can be affected by gut microbiota [3,5]. For example, gut microbe dysbiosis of pre-weaning calves hinders growth, and fecal microbiota transplantation from healthy donors can improve the growth performance of calves with diarrhea by altering the gut microbiota [6]. On the other hand, catabolites of gut microbiota can also affect health and growth by regulating the gut function and energy metabolism [7]. For example, gut microbiota-derived lipopolysaccharide (LPS) can increase the inflammatory response and oxidative stress in dairy cows [8], which can be attenuated by feeding the diet supplemented with prebiotics [9]. Recent studies have suggested that prebiotics promote the growth of pre-weaning calves by regulating the composition and community of gut microbiota [10,11]; thus, they have been supplemented to diets of dairy calves during the pre-weaning period to improve the growth performance and enhance resistance to disease [3].

Beta 1–3, 1–4 glucan, referred to as β-glucan, is one of the cell-wall polysaccharides of oat and other cereals, and is a soluble fiber [12]. It has been used in human diets and is regarded as a prebiotic due to in its beneficial role in immunomodulation and metabolic syndrome [13]. On the other hand, β-glucan has been added to animal feed for its ability to promote growth performance and increase antioxidative ability [14,15]. Ma et al. [16] found that dietary supplementation of 75 mg/kg β-glucan increased average daily gain (ADG) and gain-to-feed ratios in calves during the milk-feeding period. Studies have reported that milk replacer supplemented with 2g of β-glucan per day are beneficial to health and growth in preweaning calve, increasing the abundance of *Alloprevotella* and *Holdemanella* in gut [17]. However, the relationship of gut microbiota-host metabolism and β-glucan supplementation in pre-weaning dairy calves remains unclear.

In the present study, we sought to determine whether β-glucan affects growth and health in preweaning dairy calves by regulating the gut microbiota and host metabolism. The fecal microbiome of dairy calves that were fed oat β-glucan supplementation was analyzed by 16S rDNA amplicon sequencing, and plasma metabolite analysis was performed using liquid chromatography–tandem mass spectrometry. The fecal microbiome, plasma metabolites, and serum markers were integrated to investigate the mechanism of β-glucan. The findings will contribute to a better understanding of oat β-glucan in growth and health.

## 2. Materials and Methods

### 2.1. Animal Management

The experiment was carried out on a modern dairy farm in Sichuan Province, China, with approximately 80 dairy calves, 280 heifers, and 900 adult dairy cows. After feeding 4 L of maternal colostrum for the first 2 h after birth, all the newborn dairy calves were allocated to calf hutches individually and had free access to fresh water. The calves were bucket-fed milk replacer (Schils, Sittard, Nederland) twice daily at 0700 and 1700 pre-weening with following programs during: 4 L/d from d 2 to d 7, 5 L/d from d 8 to d 21, 6 L/d from d 22 to d 56, and 3 L/d from d 57 to d 60. The starter feed was offered on d 21 after birth. All dairy calves were weaned at on d 60 and were subsequently transferred to free-stall pens. The calves were weighed on d 1 and d 60, respectively.

### 2.2. Experimental Treatments and Sample Collection

All procedures were performed strictly according to the Laboratory Animal Care Recommendations for the Care and Use of Laboratory Animals of Sichuan Agricultural University. Fourteen Holstein newborn female dairy calves with similar birth weight (BW) were randomly divided into a control group (*n* = 7, BW = 37.43 ± 2.38 kg) and a β-glucan group (*n* = 7, BW= 37.14 ± 2.42 kg). The health status of calves was monitored in first week, of which the method of assessment was described in Love et al. [18] and Mahendran et al. [19]. Healthy dairy calves were fed milk replacer supplemented with 0 (control group) and 75 mg (β-Glucan group) oat β-Glucan kg^−1^ from d 8 after birth, and continuously fed for 14 d. The oat β-Glucan (88.69% purity on an air-dry basis; batch number SF-OGBO-210616) was purchased from Baixing Biological Technology Co., Ltd., Shangdong, China. Blood of calves were collected via the jugular vein on the d 22 and stool samples were synchronously collected via the rectum. Serum and plasma (heparin sodium as an anticoagulant) samples were collected and centrifugation at 1500× *g* for 10 min at 25 °C. All samples were stored at −80 °C until use.

### 2.3. Serum Analysis

Concentrations of serum total protein (TP), globulin (GLB), albumin (ALB), malondialdehyde (MDA), and lipopolysaccharide (LPS) were determined using commercially available kits from Nanjing Jiancheng Bioengineering Institute, China. Serum activities of glutathione (GSH), superoxide dismutase (SOD), and diamine oxidase (DAO) were also measured.

### 2.4. Fecal Microbiome Analysis

DNA was extracted from the stool samples using the TIANamp Stool DNA Kit (TIANGEN BIOTECH, Beijing, China). Amplification of 16S rDNA V3–V4 region was performed by PCR (T100 Thermal Cycler; Bio-Rad, California, USA), and sequencing of these amplicons was performed using MiSeq platform (HiSeq2500; Illumina, San Diego, CA, USA). The PCR reaction condition and sequencing-library preparation were described in the previous study [20]. Sequencing-read was analyzed by UPARSE pipeline, and sequences with >97% similarity were assigned to the same operational taxonomic units (OTU). Table of OTU was summarized at the phylum and genus levels based on the annotation.

### 2.5. Plasma Metabolites Analyses

Plasma samples were analyzed using an ultra-high-performance liquid chromatography system (1290 Infinity II, Agilent Technologies, Santa Clara, CA, USA) coupled with time-of-flight mass spectrometry (TOF 6600, AB SCIEX, Framingham, MA, USA). The analysis conditions and equipment parameters were described in our previous study [21]. Metabolic-profiling data processing was performed to obtain the retention time and peak area using XCMS software [22]. The plasma metabolites were identified by matching an in-house standard database from Shanghai Applied Protein Technology Co. Ltd., Shanghai, China [23,24].

### 2.6. Statistical Analyses

Data were analyzed using R (version 4.2; https://www.r-project.org (accessed on 5 May 2022)) and SAS (version 9.4; SAS Institute Inc., Cary, NC, USA) software. Two-tailed Student’s *t*-tests were performed to compare variables between control and β-glucan groups. The analyses of origin of metabolites and Kyoto Encyclopedia of Genes and Genomes (KEGG) pathway were performed using MetOrigin online platform [25]. After the OTU table was rarified and scaled, alpha diversity analyses were conducted using MicrobiomeAnalyst [26]. Nonmetric multidimensional scaling (NMDS) analysis based on Bray–Curtis distance was performed to compare the heterogeneous community structure of gut microbiota between control and β-glucan groups. To analyze the association of fecal microbiota and plasma metabolites, multivariate correlation analysis was performed using 3MCor [27], including two-way orthogonal partial least-squares (O2PLS) analysis, co-inertia analysis (CIA), and correlation network analysis. Correlations between serum variables and microbiota/metabolites were assessed by Spearman’s rank correlation coefficient. Significance was defined as *p* < 0.05 and a trend was defined as 0.05 < *p* < 0.1. Data are presented as a mean ± SD.

## 3. Results

### 3.1. Serum Markers and Body Weight Analyses

**Figure 1:**
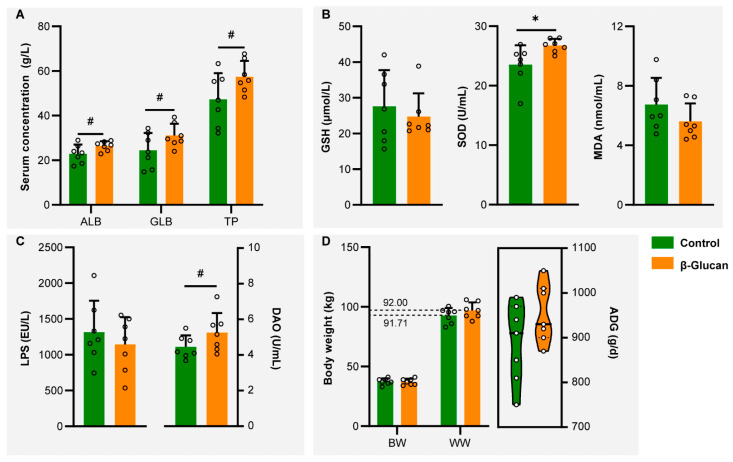
Changes in serum markers and body weights of dairy calves between the control and β-glucan groups. (**A**) Total protein (TP), globulin (GLB), and albumin (ALB) concentrations in serum that differed between the control and β-glucan groups. (**B**) Changes in antioxidant levels in dairy calf with β-glucan supplementation, including glutathione (GSH), superoxide dismutase (SOD), and malondialdehyde (MDA). (**C**) Serum lipopolysaccharide (LPS) and diamine oxidase (DAO) levels differed between the control and β-glucan groups. (**D**) Changes in birth weight (BW), weaning weight (WW) and average daily gain (ADG) between the control and β-glucan groups. Data are presented as mean ± SD. 0.05 < ^#^ *p* < 0.10, * *p* < 0.05.

Serum ALB, GLB, and TP concentrations were higher (*p* < 0.1) in the β-glucan group than those in the control group (Figure 1A). In contrast to the control group, serum SOD activity of dairy calves markedly (*p* < 0.05) increased in the β-glucan group (Figure 1B). The GHS and MDA levels did not differ between control and β-glucan groups. Compared with the control group, DAO activity of calves increased (*p* < 0.1) in the β-glucan group (Figure 1C). However, LPS concentration in serum did not differ between groups. The ADG of calves in the control and β-glucan groups from birth to weaning were 890 g/d and 950 g/d, respectively (Figure 1D). The mean weaning weight (WW) in the β-glucan group was 4.29 kg heavier than that of the control group.

### 3.2. Alterations of Gut Microbiota in Dairy Calves

**Figure 2:**
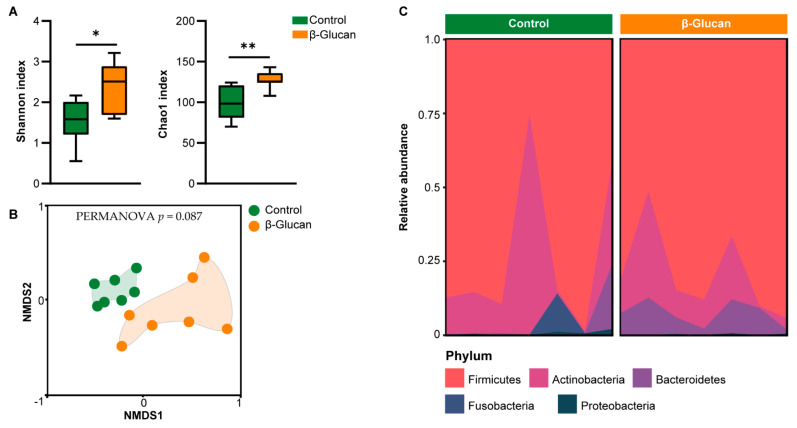
Effects of β-glucan on the composition of gut microbiota in dairy calves. (**A**) Box plot outlining the α-diversity of gut microbiota between the control and β-glucan groups as per the Shannon or Chao 1 index. (**B**) Nonmetric multidimensional scaling (NMDS) analysis of gut microbiota between the control and β-glucan groups based on the permutational multivariate analysis of variance. (**C**) Area plot indicates the relative abundance of gut microbiota at phylum level. * *p* < 0.05, ** *p* < 0.01.

**Figure 3:**
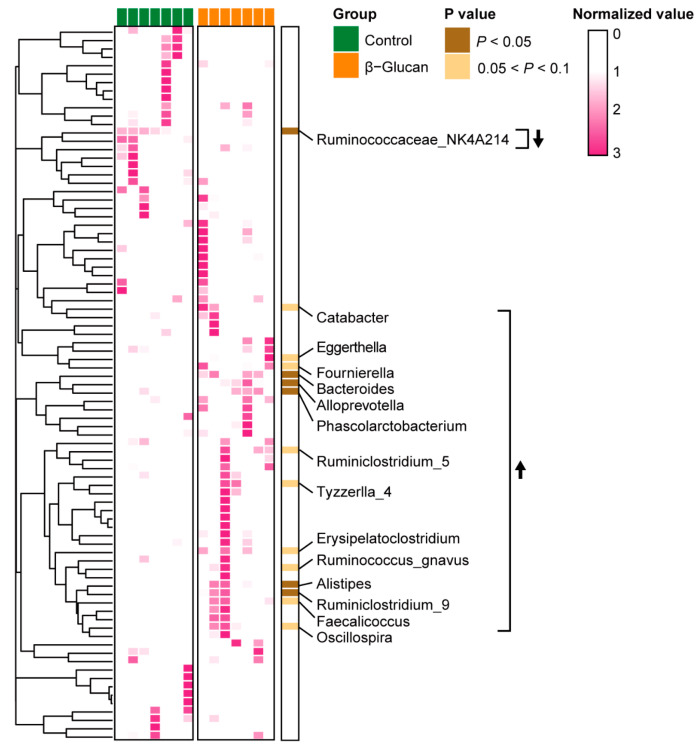
Heatmap of genera taxa in stool of the dairy calves. Only differential genera with *p* < 0.1 are shown. The upward arrow indicates that the genus level was increased in the β-glucan group than the control group, and the downward arrow indicates that the genus level was decreased in the β-glucan group.

Dairy calves with β-glucan supplement had higher Shannon and Chao1 indexes in alpha diversity compared with those in the control group (Figure 2A), indicating that the β-glucan increased gut microbiota richness of calves. NMDS analysis showed that the heterogeneous community structure in gut microbiota differed (PERMANOVA *p* = 0.087) between the control and β-glucan groups (Figure 2B). Firmicutes, Actinobacteria, and Bacteroidetes were the major taxa in the gut of per-weaning dairy calves (Figure 2C). The abundance of Firmicutes and Actinobacteria had no differences between the control and β-glucan groups. However, the Firmicutes-to-Bacteroidetes ratio was lower (*p* = 0.09) in the β-glucan group than that in the control group. Eighty-four genera taxa were found in feces of dairy claves. Compared with the control groups, the abundance of *Bacteroides*, *Alloprevotella*, *Phascolarctobacterium*, and *Alistipes* were markedly increased (*p* < 0.05) in the β-glucan group (Figure 3). The abundance of *Oscillospira*, *Ruminclostridum_5*, *Ruminococcus_gnavus*, and *Faecalicoccus* was increased (*p* < 0.1) in the β-glucan group calves. However, *Ruminococcaceae_NK4A214* abundance was lower in the β-glucan group when compared to the control group.

### 3.3. Alterations of Plasma Metabolites in Dairy Calves

**Figure 4:**
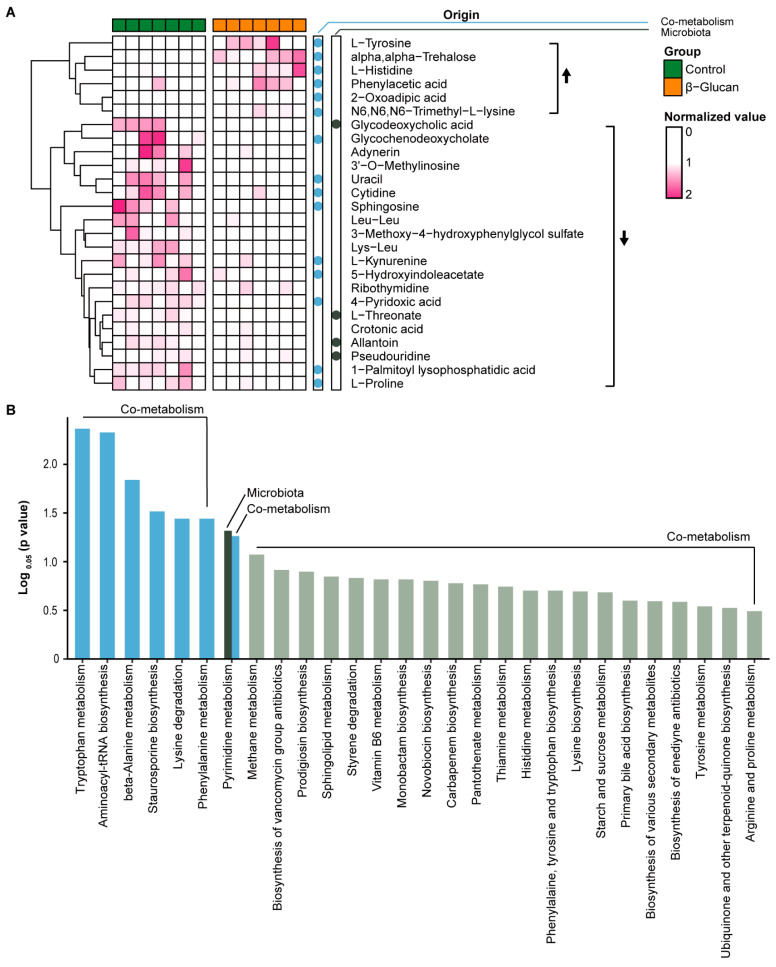
(**A**) Heatmap indicates the differential plasma metabolites between the control and β-glucan groups. Solid dot indicates the different origins of the metabolites, including microbiota and co-metabolism. The upward arrow indicates that the metabolite levels were higher in the β-glucan group than those in the control group, and the downward arrow indicates that the metabolite levels were lower in the β-glucan group. (**B**) Bar plot of the KEGG pathway analysis of different metabolites.

A total of 282 plasma metabolites were identified with a metabolomics standards initiative level 1 or 2 identification. Twenty-six differential (*p* < 0.05) metabolites were identified between the control and β-glucan groups, twenty of which were decreased in the β-glucan group (Figure 4A). These metabolites were mainly derived from the common metabolism (co-metabolism) of host and gut microbiota, such as kynurenine, 5-hydroxyindoleacetate, 1-palmitoyl lysophosphatidic acid, sphingosine, 4-pridoxic acid, and glycochenodeoxycholate. Of notes, some metabolites were derived from the gut microbiota catabolism, including glycodeoxycholic acid, L-threonate, allantoin, and pseudouridine. KEGG pathway analysis indicated that aromatic amino acids metabolism was markedly changed between the control and β-glucan groups, including tryptophan metabolism and phenylalanine metabolism (Figure 4B). Moreover, pyrimidine metabolism, which belongs to the microbiota or co-metabolism, was changed in β-glucan supplemented calves.

### 3.4. Association of Gut Microbiota and Plasma Metabolites

**Figure 5:**
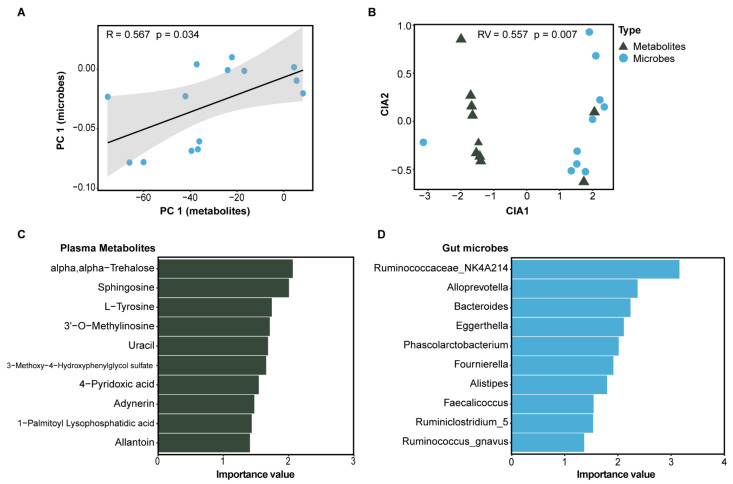
Multidimensional analysis of the association between gut microbiota and plasma metabolites. (**A**) Two-way orthogonal partial least-squares (O2PLS) analysis between gut microbes and plasma metabolites. *X*-axis represents the first principal component of metabolites, and *Y*-axis represents the first principal component of microbes. (**B**) Co-inertia analysis (CIA) of the relationship between microbiota and metabolites. (**C**,**D**) show the top 10 metabolites and microbes screened based on CIA, respectively.

Correlation analysis of O2PLS showed strong correlations (R = 0.567 and *p* = 0.034) between changes in the gut microbiota and levels of plasma differential metabolites (Figure 5A). Moreover, CIA of the genera taxa abundance and the plasma metabolites revealed a close relationship between them (RV coefficient = 0.557 and *p* = 0.007, Figure 5B). The key microbiota and its associated metabolites were screened based on CIA (Figure 5C,D). The covariance between the genera taxa and metabolites showed that the change in plasma metabolites were associated with *Ruminococcaceae_NK4A214*, *Alloprevotella*, *Bacteroides*, *Phascolarctobacterium*, *Fournierella*, and *Alistipes*.

### 3.5. Association among Gut Microbe, Plasma Metabolite, and Clinical Phenotype

**Figure 6:**
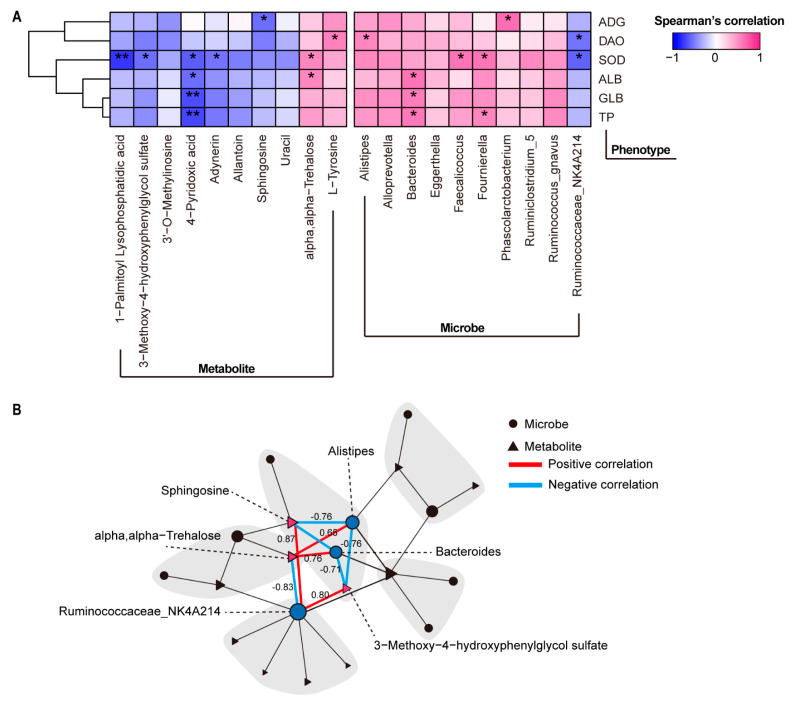
Association among gut microbe, plasma metabolite, and clinical phenotype. (**A**) Heatmap plot shows the correlation between gut microbiota or plasma metabolites and clinical parameters base on Spearman correlation. (**B**) Networks between gut microbes and plasma metabolites. Node represents key metabolites or microbes, the size of which is determined by the centrality, and solid lines represents the correlation coefficients between microbes and metabolites. * *p* < 0.05, ** *p* < 0.01.

The levels of serum markers differed between the control and glucan groups, including SOD, ALB, GLB, TP, and DAO. To further investigate the relationships between clinical phenotype and microbiota/metabolite, 10 genera and 10 metabolites that correlated with serum markers and ADG were performed using Spearman’s rank correlation test (Figure 6A). The increase in activity of serum SOD was positively correlated with the increase in *Fournierella* and *Faecalicoccus* abundances, and was negatively correlated with the decrease in the plasma 1-palmitoyl lysophosphatidic acid and 3-methoxy-4-hydroxyphenylglycol sulfate (MHPG-SO4) levels. The increase in DAO level was negatively correlated with the decrease in abundance of *Ruminococcaceae_NK4A214*, and was positively correlated with the increase in tyrosine level. The ADG was negatively correlated with plasma sphingosine level, and was positively correlated with *Phascolarctobacterium*. Furthermore, the 4-pyridocix acid and *Bacteroides* were strongly correlated with the three serum makers, including ALB, GLB, and TP. Notably, the three genera were closely correlated with the three metabolites in alteration of serum markers (Figure 6B). The abundance of *Ruminococcaceae_NK4A214* was positively correlated with MHPG-SO4 and sphingosine levels. However, the abundance of *Alistipes* was negatively correlated with MHPG-SO4 and sphingosine levels. The abundance of *Bacteroides* was positively correlated with the alpha, alpha-trehalose level, and was negatively correlated with the MHPG-SO4 level.

## 4. Discussion

The growth and health of dairy calves in the pre-weaning period are closely related to the future production performance, especially the early milk-feeding phase [28]. Nutrition is a major factor affecting the growth and health of dairy calves [29], and serum TP and ALB concentrations have been used to evaluate the nutritional status [30,31]. In the current study, we found that serum TP and ALB levels were increased in the dairy calves that were fed with oat β-glucan supplementation. A previous study also reported that dietary β-glucan supplementation was associated with the increased serum TP and ALB levels, further promoting the growth rates [32]. Consistent with our findings, Ma et al. [16] reported that β-glucan improved the ADG and WW of calves. In addition, β-glucan is regarded as a functional food which was beneficial to improve immunity and antioxidant ability [33]. We found that the globulin and SOD levels were higher in the β-glucan group than those in the control group. Abo Ghanima et al. [34] reported that serum GLB and SOD levels were increased due to β-glucan administration in New Zealand White weanling rabbits. Previous studies have shown that β-glucan positively regulates immunity via the activation of Dectin-1 receptors [35]. Yu et al. [36] found that β-glucan increased the activity of SOD and decreased the production of reactive oxygen species under the LPS stimulation, thereby alleviating oxidative stress via the Dec-tin-1/Nrf2/HO-1 signaling pathway. Previous studies have shown that β-glucan increases milk yield and reduces milk somatic cell count of postpartum dairy cows by enhancing antioxidative capacity and reducing inflammatory response [14]. Therefore, the consumption of β-glucan can promote the production performance of dairy cows, but the effect of prolonged β-glucan supplementation on the profitability of dairy calves needs to be further studied.

The milk-feeding period is a key phase of gut development in dairy calves, in which nutritional regulation of gut health is crucial for a successful transition to weaning phase [2]. Gut barrier dysfunction in early weaning period limits to the future growth in dairy calves [37]. Circulating DAO and LPS levels have been used as markers to assess gut barrier function [38]. Fukuda et al. [39] found that the plasma DAO activity markedly decreased in calves with diarrhea, but the parenteral nutrition treatment could increase DAO activity in calves with diarrhea [40]. In the current study, we found that the β-glucan supplementation increased the DAO activity in pre-weaning dairy calves, which may suggest that β-glucan can promote the gut barrier function. There is increasing evidence that the gut microbiota and their catabolites are closely related to gut health [41]. The composition and structure of the gut microbiota alters in the dysfunctional gut barrier, for example, the abundance of Firmicutes increased and the abundance of Bacteroidetes decreased in intestinal inflammation [42]. A recent study found that β-glucan improved gut barrier by decreasing the Firmicutes/Bacteroidetes ratio [42], which is consistent with our findings. In addition, we found that β-glucan increased the abundances of *Bacteroides*, *Alloprevotella*, *Fournierella*, *Alistipes*, and *Oscillospira* and decreased the abundance of *Ruminococcaceae_NK4A214* in feces of the dairy calves. *Bacteroides*, *Alistipes*, and *Alloprevotella* belonging to Bacteroidetes, and they contribute to the production of short-chain fatty acids (SCFAs) and further benefit gut health [43]. Beta-glucan is a soluble fiber that can be fermented by the microbiota and produce SCFAs in the gut [40,42]. Similar to Bacteroides, *Ruminococcaceae_NK4A214* is a butyrate producer, which plays a positive role in reducing disease risk [44]. However, a recent study showed that the abundance of *Ruminococcaceae_NK4A214* was increased in dairy goats with subacute ruminal acidosis and was positively correlated with LPS level [45]. Furthermore, in line with our findings, Wu et al. [46] suggested that β-glucan promoted growth performance during the weaning phase by improving gut health and increasing the abundance of *Fournierella* and *Oscillospira*. Therefore, whether β-glucan alters the production of SCFAs by regulating the intestinal flora and thus plays a positive role in growth and health remains to be further investigated.

Besides gut health, gut microbiota is also closely related to host metabolism [47]. Joyce et al. suggested [48] that oat β-glucan modulated cholesterol metabolism and altered bile acid composition through altering bile salt hydrolase (BSH) activity of the gut microbiota; for instance, *Bacteroides* and *Lactobacillus* are BSH-producing bacteria. Consumption of β-glucan has been shown to reduce host cholesterol levels by reducing the amount of circulating bile acid [48,49]. Consistent with previous studies, we found that consumption of β-glucan decreased bile acid levels in plasma of dairy calves, including glycodeoxycholic acid and glycochenodeoxycholate. In addition, we found that the levels of kynurenine and 5-hydroxyindoleacetate in the β-glucan group were lower than those in the control group. Kynurenine is derived from tryptophan via the indoleamine 2,3-dioxygenase (IDO) or tryptophan 2,3-dioxygenase (TDO) pathways [50]. Proinflammatory cytokines, including interleukin-1β and tumor necrosis factor-α, increase IDO and TAO activities, resulting in the increase of kynurenine level [51]. Serotonin is a neurotransmitter derived from tryptophan metabolism that produces 5-hydroxyindoleacetate via the monoamine oxidase (MAO) pathway [52]. Similar to IDO, MAO activity is enhanced in inflammatory status [53]. Van der Leek et al. [54] found that gut microbiota played an important role in regulating the function of the kynurenine-IDO pathway. Of note, in the present study, β-glucan increased the trehalose level, which had been reported to protect against oxidative stress and inflammation response by regulating the Keap1–Nrf2 pathway [55]. We also found that β-glucan supplementation decreased plasma MHPG-SO4 and sphingosine levels. As a major metabolite of norepinephrine, the high level of MHPG-SO4 is related to the development of eating disorders [56]. Similar to MHPG-SO4, sphingosine was derived from palmitic acid metabolism in vivo, and serum sphingosine levels have been reported to be higher in eating-related disorders [57,58]. Therefore, β-glucan may contribute to the health of dairy calves by positively regulating the interactions of gut microbes with the host.

## 5. Conclusions

The pre-weaning period is a key phase for the growth and development of dairy calves. Supplementation of the β-glucan in the diet increased serum TP, ALB, GLB, SOD, and DAO levels. The alterations in the abundance of gut microbiota and the plasma metabolic profile were correlated with the increases in serum markers. We therefore propose that the β-glucan plays a beneficial role in the interaction between the gut microbe and the host and may serve as a potential prebiotic in dairy-calf feeding. Further studies on fecal metagenome and metabolites are encouraged to investigate the function of gut microbes.

## Data Availability

Not applicable.
